# Supplementary material for: Predicting visual function by interpreting a neuronal wiring diagram
Source: Nature. 2024 Oct 2;634(8032):113–23. doi: 10.1038/s41586-024-07953-5 (PMC11446822; doi:10.1038/s41586-024-07953-5)
Supplement: Supplementary file 6 — CRF and ERF predictions for individual TmY4 and TmY9 cells. Analogous to Supplementary Data 3, but for TmY target types. Shown are the top four monosynaptic pathways, the strongest pathway passing through each of the top ten intermediary types (ranking from Extended Data Fig. 7), and the trisynaptic pathway Tm1–TmY–Dm3–TmY (see the section entitled Prediction of spatial normalization). [file 41586_2024_7953_MOESM6_ESM.zip › DataS4/TmY4/720575940607898204.pdf]

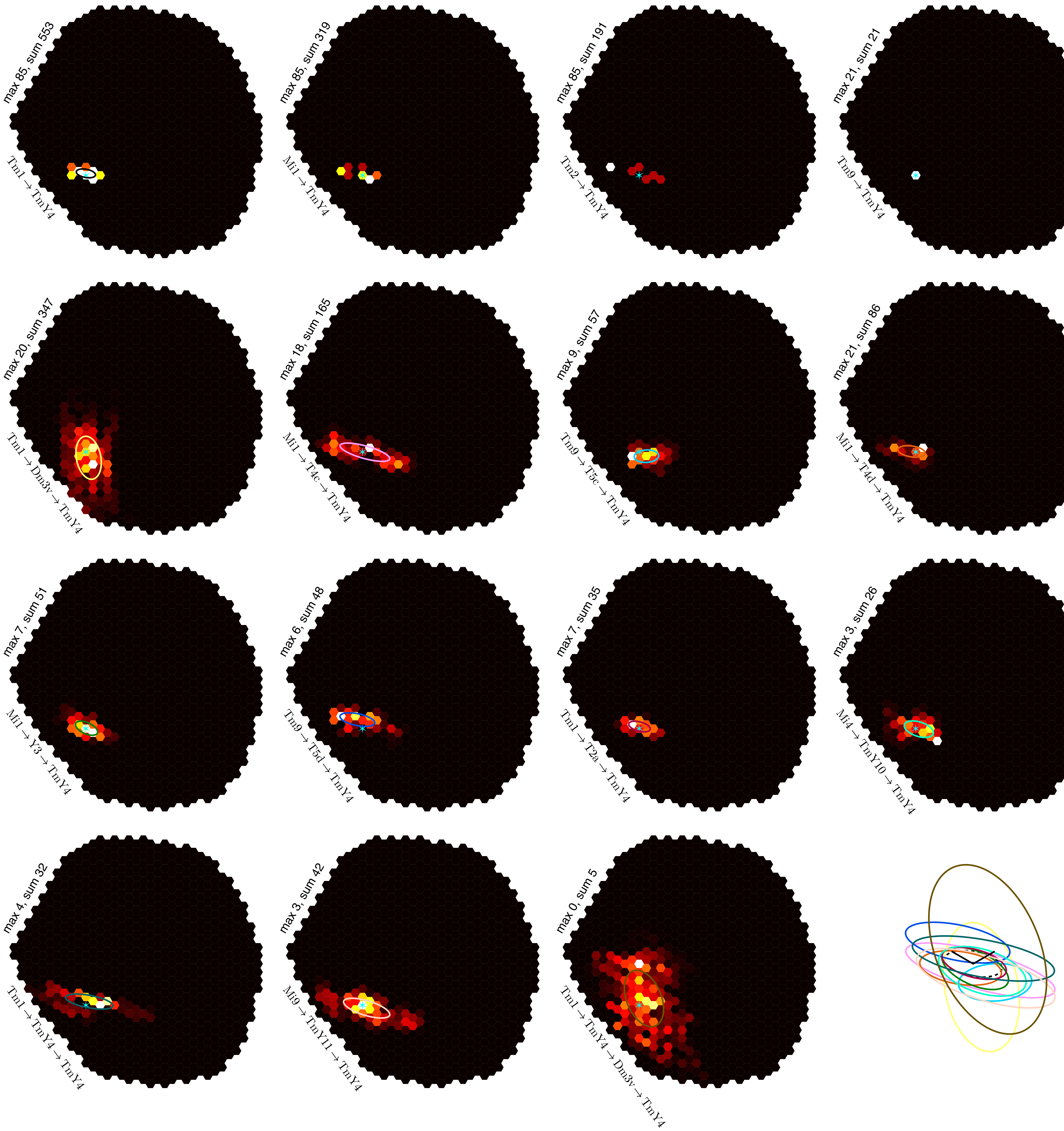

max 85, sum 553
Tm1 → TmY4
max 85, sum 319
Mi1 → TmY4
max 85, sum 191
Tm2 → TmY4
max 21, sum 21
Tm9 → TmY4
max 20, sum 347
Tm1 → Dm3v → TmY4
max 18, sum 165
Mi1 → T4c → TmY4
max 9, sum 57
Tm9 → T5c → TmY4
max 21, sum 86
Mi1 → T4d → TmY4
max 7, sum 51
Mi1 → Y3 → TmY4
max 6, sum 48
Tm9 → T5d → TmY4
max 7, sum 35
Tm1 → T2a → TmY4
max 3, sum 26
Mi4 → TmY10 → TmY4
max 4, sum 32
Tm1 → TmY4 → TmY4
max 3, sum 42
Mi9 → TmY11 → TmY4
max 0, sum 5
Tm1 → TmY4 → Dm3v → TmY4
